# Radiation-induced malignancies following radiotherapy for breast cancer

**DOI:** 10.1038/sj.bjc.6602084

**Published:** 2004-08-03

**Authors:** R Roychoudhuri, H Evans, D Robinson, H Møller

**Affiliations:** 1 Thames Cancer Registry, Division of Cancer Studies, Guy's, King's and St. Thomas' School of Medicine, 1st Floor, Capital House, 42 Weston Street, London SE1 3QD, UK

**Keywords:** radiotherapy, breast cancer, second malignancy, radiation

## Abstract

With advances in diagnosis and treatment, breast cancer is becoming an increasingly survivable disease resulting in a large population of long-term survivors. Factors affecting the quality of life of such patients include the consequences of breast cancer treatment, which may have involved radiotherapy. In this study, we compare the incidence of second primary cancers in women who received breast radiotherapy with that in those who did not (non-radiotherapy). All women studied received surgery for their first breast cancer. Second cancers of the lung, colon, oesophagus and thyroid gland, malignant melanomas, myeloid leukaemias and second primary breast cancers were studied. Comparing radiotherapy and non-radiotherapy cohorts, elevated relative risks (RR) were observed for lung cancer at 10–14 years and 15 or more (15+) years after initial breast cancer diagnosis (RR 1.62, 95% confidence interval [CI] 1.05–2.54 and RR 1.49, 95% CI 1.05–2.14, respectively), and for myeloid leukaemia at 1–5 years (RR 2.99, 95% CI 1.13–9.33), for second breast cancer at 5–10 years (RR 1.34, 95% CI 1.10–1.63) and 15+ years (RR 1.26, 95% CI 1.00–1.59) and oesophageal cancer at 15+ years (RR 2.19, 95% CI 1.10–4.62).

With recent advances in diagnosis and treatment, breast cancer is becoming an increasingly survivable disease resulting in a large population of long-term survivors. Clearly, factors affecting the quality of life and length of survival of such patients include the consequences of breast cancer treatment, which may have involved radiotherapy. There is copious evidence for the link between radiation exposure and carcinogenesis, especially from the epidemiological study of survivors of atomic bomb irradiation (Land *et al*, 2003; Preston *et al*, 2003) and it has been suggested that irradiation of surrounding tissues during breast radiotherapy can, through similar mechanisms, cause second cancers to develop within these tissues (Harvey and Brinton, 1985; Neugut *et al*, 1999). While the benefits of radiotherapy outweigh the risks of developing subsequent cancers, the presence of such risks could implicate the need for further investigation into methods of minimising the radiation dose delivered to surrounding tissues or the volume of such tissues exposed.

Several different factors may contribute to the co-occurrence of multiple primary cancers in an individual besides radiation-induced oncogenesis: genetic predisposition, environmental exposure to carcinogens, reproductive factors, misdiagnosis of metastases as primary cancers, increased medical surveillance following the first cancer and other treatment-related effects. Temporal patterns underlying associations in the co-occurrence of cancers are useful in discerning the predominant aetiology underlying these associations. For example, genetic and environmental predisposition would be expected to cause a general, temporally nonspecific increase in the incidence of second cancers, while iatrogenic tumours resulting from the side effects of treatment for a first cancer would be expected to develop some time after this treatment. Indeed, Boice *et al* (1996) have suggested that cancers resulting from an exposure to radiation would develop within predictable time windows: after 10 years for solid tumours and within 5 years for leukaemias.

In this paper, we have used one of the world's largest population-based cancer registries to compare the incidence of certain second cancers in patients who either have, or have not, received radiotherapy alongside surgery for breast cancer. Cancers of the lung, colon, oesophagus and thyroid gland, along with malignant melanomas of the skin, myeloid leukaemias and second primary breast cancers, were analysed, since these neoplasms have been previously associated with radiotherapy of the breast (Harvey and Brinton, 1985; Evans *et al*, 2001; Deutsch *et al*, 2003; Smith *et al*, 2003).

## MATERIALS AND METHODS

The Thames Cancer Registry (TCR) database was used to identify breast cancer patients who either had or had not received radiotherapy alongside surgery for breast cancer, and who went on to develop further primary cancers. The Thames Cancer Registry is a population-based registry which collects data on cancer in residents of South East England (currently a population of 14 million). Data collection began in 1960 in the South Thames Region and was extended in 1985 to also cover the North Thames Region. The patients registered at the TCR represent a cohort of individuals followed up from diagnosis to death, and the database currently contains over 1.5 million incident cancers.

In the analysis of multiple cancers, it is important that metastases or recurrences of the initial tumour should not be misclassified as primary tumours. The rules for accepting a second tumour as a new primary rather than a metastasis are well defined and nationally agreed among the UK cancer registries. As a general rule, a new primary tumour needs to be at a different anatomical site and of a different histological type from the first tumour, or to be stated explicitly as being a new tumour by the treating clinician.

Index cohorts were created by extracting all registrations of females with a first breast cancer who received surgery for this breast cancer, resident in the North or South Thames region and diagnosed between 1 January 1961 and 31 December 2000 from the TCR database. Those who also received radiotherapy for this breast cancer were placed into the RT cohort, while those who did not were placed into the non-RT cohort, and analyses were performed separately on these subgroups. Women who received additional treatment (e.g. chemotherapy) for their primary breast cancer were excluded from the study. For women diagnosed with two or more cancers subsequent to breast cancer, only the first subsequent cancer was considered, since if multiple cancers were considered treatments for these cancers themselves might cause additional effects on the incidence of subsequent cancers.

Second cancers occurring within 1 year of the initial cancer, at the same site, with the same laterality and histology were excluded, as were patients with a missing date of birth and cancers without a year of diagnosis or without information on the location of residence of the patient at the time of diagnosis. Patients with two cancers at different sites diagnosed on the same day were also excluded from the analysis.

To obtain standardised incidence ratios of subsequent cancers following diagnosis of breast cancer, we computed person-years at risk in each cohort and applied appropriate population-based cancer incidence rates. For a given subsequent cancer site, person-years at risk were calculated from the date of diagnosis of breast cancer to the date of first diagnosis of cancer at the specified site or to the exit date (date of death, loss to follow up or 85th birthday, whichever was earlier). Patients diagnosed prior to 1 January 1971 were followed up actively, obtaining death information, until 31 December 1982. These were censored at this date. Patients diagnosed after 1 January 1971 were followed up through the NHS Central Register, which provides notification of all deaths routinely to the registry. Age- and period-specific cancer incidence rates for the region covered by the TCR were then applied to the cohort for intervals following breast cancer diagnosis of less than 1 year, 1–4, 5–9, 10–14 and 15 or more (15+) years to calculate the number of subsequent tumours that would be expected for each site in each specific interval. This number was compared with the observed number to obtain a standardised incidence ratio (SIR) estimate, and exact 95% confidence intervals were calculated assuming an underlying Poisson distribution. Relative risks (RRs) were calculated by comparison of the SIR in the RT and non-RT cohorts, and exact 95% confidence intervals calculated using the method described by Breslow and Day (1982). Calculations were performed using the statistical package Stata (StataCorp, 1999). The following cancer sites were studied, classified according to the 10th revision of the International Classification of Diseases (ICD) (World Health Organisation, 1992): lung and bronchus (C34) referred to in this paper as simply lung cancer, breast (C50), colon (C18), oesophagus (C15), malignant melanoma of the skin (C43), thyroid (C73) and myeloid leukaemia (C92).

## RESULTS

A total of 64 782 women who had received surgery following a first cancer of the breast were included in this study. Of these, 33 763 received radiotherapy in addition to surgery for this cancer (RT cohort), and 31 019 did not (non-RT cohort). A total of 5217 second primary tumours were detected, and 2857 of these second tumours were at one of the sites of interest.

Results are tabulated by interval of follow-up and second cancer site in Table 1

**Table 1:** Standardised incidence ratios and RR of developing second cancer for women who received surgery and radiotherapy for breast cancer (RT), compared with those who received surgery only (non-RT), tabulated by second cancer site and interval of follow-up

	**Nonradiotherapy cohort**				**Radiotherapy cohort**					
**Interval (years)**	**Observed no. of tumours**	**Expected no. of tumours**	**SIR**	**95% CI**	**Observed no. of tumours**	**Expected no. of tumours**	**SIR**	**95% CI**	**Relative Risk**	**95% CI**
*Lung*										
0–	27	22.8	1.18	0.78–1.72	10	21.1	0.47	0.23–0.87	0.40	0.17–0.85
1–	50	79.4	0.63	0.47–0.83	50	77.4	0.65	0.48–0.85	1.03	0.68–1.55
5–	60	77.5	0.77	0.59–1.00	69	81.6	0.85	0.66–1.07	1.09	0.76–1.57
10–	34	56.0	0.61	0.42–0.85	62	63.0	0.98	0.75–1.26	1.62	1.05–2.54
15+	55	55.9	0.98	0.74–1.28	80	54.5	1.47	1.16–1.83	1.49	1.05–2.14
										
*Myeloid Leukaemia*										
0–	2	2.0	1.02	0.11–3.67	2	1.8	1.13	0.13–4.08	1.11	0.08–15.3
1–	6	6.8	0.89	0.32–1.93	17	6.4	2.66	1.55–4.25	2.99	1.13–9.33
5–	7	6.5	1.08	0.43–2.23	8	6.7	1.19	0.51–2.35	1.10	0.35–3.59
10–	5	4.6	1.09	0.35–2.55	3	5.1	0.59	0.12–1.72	0.54	0.08–2.78
15+	2	4.5	0.45	0.05–1.62	9	4.3	2.09	0.95–3.96	4.66	0.97–44.8
										
*Breast*										
0–	99	56.6	1.75	1.42–2.13	78	58.7	1.33	1.05–1.66	0.76	0.56–1.03
1–	266	186.4	1.43	1.26–1.61	248	200.5	1.24	1.09–1.40	0.87	0.73–1.03
5–	173	167.5	1.03	0.88–1.20	264	190.7	1.38	1.22–1.56	1.34	1.10–1.63
10–	151	113.1	1.34	1.13–1.57	158	132.6	1.19	1.01–1.39	0.89	0.71–1.12
15+	138	101.6	1.36	1.14–1.60	170	99.5	1.71	1.46–1.99	1.26	1.00–1.59
										
*Oesophagus*										
0–	5	3.9	1.29	0.41–3.00	1	3.3	0.31	0.00–1.71	0.24	0.00–2.11
1–	11	13.5	0.81	0.41–1.46	13	12.1	1.07	0.57–1.83	1.32	0.55–3.25
5–	18	13.3	1.35	0.80–2.14	14	13.3	1.05	0.57–1.76	0.78	0.36–1.66
10–	11	9.8	1.12	0.56–2.01	11	10.9	1.01	0.50–1.81	0.90	0.35–2.29
15+	13	10.2	1.28	0.68–2.18	28	10.0	2.80	1.86–4.05	2.19	1.10–4.62
										
*Malignant melanoma of the skin*										
0–	4	2.7	1.51	0.41–3.86	5	3.3	1.51	0.49–3.51	1.00	0.22–5.15
1–	11	8.7	1.26	0.63–2.26	14	11.4	1.23	0.67–2.06	0.97	0.41–2.36
5–	6	7.9	0.76	0.28–1.66	8	11.0	0.73	0.31–1.44	0.96	0.29–3.35
10–	4	5.4	0.74	0.20–1.89	8	7.9	1.01	0.44–2.00	1.37	0.37–6.20
15+	9	5.8	1.55	0.71–2.95	10	6.3	1.59	0.76–2.93	1.03	0.37–2.85
										
*Colon*										
0–	22	18.1	1.22	0.76–1.84	11	15.0	0.73	0.37–1.31	0.60	0.26–1.30
1–	48	60.7	0.79	0.58–1.05	49	53.6	0.91	0.68–1.21	1.16	0.76–1.76
5–	42	55.2	0.76	0.55–1.03	55	54.1	1.02	0.77–1.32	1.34	0.88–2.05
10–	27	36.0	0.75	0.49–1.09	27	39.6	0.68	0.45–0.99	0.91	0.51–1.61
15+	20	29.0	0.69	0.42–1.06	21	28.2	0.74	0.46–1.14	1.08	0.56–2.10
										
*Thyroid*										
0–	2	4.6	0.44	0.05–1.58	5	4.7	1.06	0.34–2.48	2.43	0.40–25.7
5–	6	3.1	1.96	0.72–4.27	4	3.4	1.18	0.32–3.01	0.60	0.13–2.56
10–	1	2.0	0.49	0.01–2.74	3	2.3	1.28	0.26–3.75	2.60	0.21–137
15+	2	1.8	1.09	0.12–3.92	5	1.8	2.78	0.90–6.48	2.56	0.41–26.3

, and results for lung cancer, myeloid leukaemia, breast cancer and oesophageal cancer are shown graphically in Figure 1

**Figure 1:**
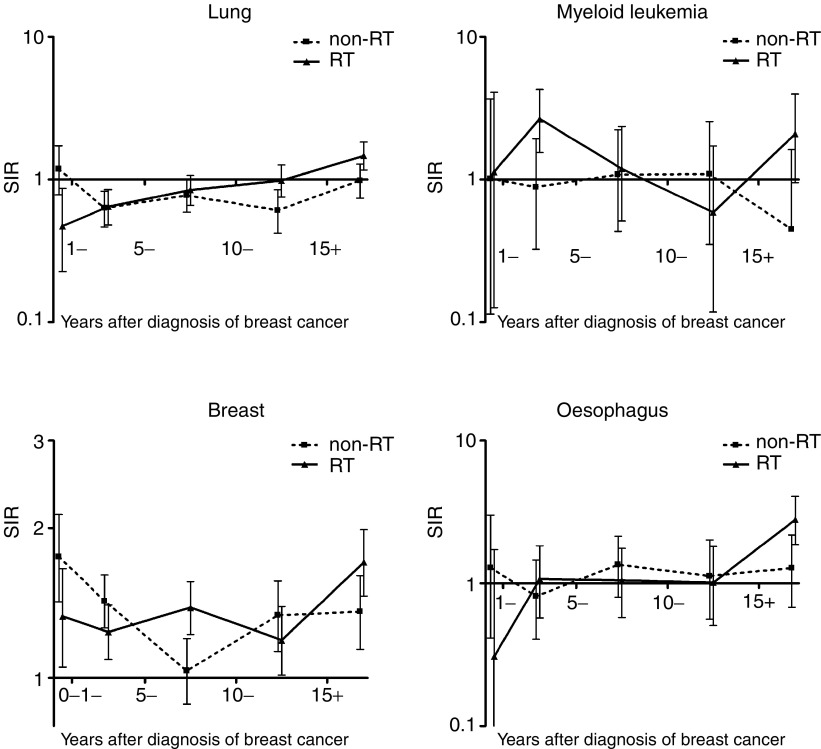
Standardised incidence ratios of lung cancer, myeloid leukaemia, breast cancer and oesophageal cancer in patients who received surgery and RT for breast cancer, and in patients who received surgery only (non-RT), plotted against interval following breast cancer diagnosis. Vertical bars are 95% confidence intervals.

.

The RR of developing lung cancer was shown to be significantly elevated in the RT cohort compared with the non-RT cohort at both 10–14 and 15 or more (15+) years after diagnosis of breast cancer (RR 1.62, 95% CI 1.05–2.54 and RR 1.49, 95% CI 1.05–2.14, respectively). The SIRs for lung cancer in the 1–5-year period of follow-up were low in both groups, and there was also found to be a decreased RR of lung cancer within the first year following diagnosis of breast cancer in the RT cohort when compared with the non-RT cohort (RR 0.40, 95% CI 0.17–0.85). A significantly increased RR of developing myeloid leukaemia was observed at 1–5 years (RR 2.99, 95% CI 1.13–9.33). There was an elevated SIR of second primary breast cancer in both the non-RT and RT cohorts, regardless of the period of follow-up. However, at 5 years of follow-up and longer, the excess was greater in the RT cohort, particularly so in the interval from 5 to 10 years (RR 1.34, 95% CI 1.10–1.63) and 15+ years (RR 1.26, 95% CI 1.00–1.59). Considered over the whole period of follow-up from 5 years and onwards, the SIRs were 1.21 and 1.40 in the RT cohort and non-RT cohort, respectively, and the overall RR was 1.16 (95% CI 1.02–1.31).

The RR of oesophageal cancers was increased significantly 15+ years after diagnosis and radiotherapy (RR 2.19, 95% CI 1.10–4.62). There were no significant differences observed in the incidence of second cancers of the colon, thyroid gland or malignant melanomas of the skin in the RT cohort when compared with the non-RT cohort in any period of follow-up.

## DISCUSSION

In this study, we have used a large population-based cancer registry to examine the effects of radiotherapy for breast cancer on the incidence of subsequent second cancers. Such epidemiological analyses, however, can be highly sensitive to biasing factors. By selecting RT and non-RT cohorts, one also consistently selects for those factors taken into account when a clinical decision is made concerning whether or not to use radiotherapy. Genetic and environmental predispositions may underlie the co-occurrence of multiple cancers in an individual, and if these predispositions also underlie other factors affecting the aforementioned clinical decisions then bias may be introduced. Only patients who had received surgery for breast cancer were included in this study rather than all first tumours of the breast. This is likely to reduce any bias between the RT and non-RT cohorts by reducing variability in the parent population. The authors of two other important epidemiological studies of a similar nature have also usefully used surgery as the background for their RT and non-RT cohorts (Boice *et al*, 1985; Brenner *et al*, 2000), as acknowledged by Hall and Wuu (2003). Boice has suggested that a certain latency period passes before tumours resulting from exposure to radiation develop, and that this period depends upon the cancer type. Leukaemias are expected to develop within 5 years, while an induced excess of solid tumours is observed after 10 or more years (Boice *et al*, 1996), and there is copious epidemiological evidence to support this (Neugut *et al*, 1993; Rubino *et al*, 2003; Zablotska and Neugut, 2003). Even if similar latencies exist for exposure to other carcinogenic breast cancer treatments, a significant excess in the incidence of cancers in the RT cohort compared with the non-RT cohort and following the expected latency period would support radiotherapy as the predominant aetiology.

A significant excess in the incidence of myeloid leukaemia 1–5 years after breast cancer diagnosis was observed in radiotherapy patients. This is consistent with previous epidemiological studies (Curtis *et al*, 1992; Smith *et al*, 2003). Smith *et al*, however, also found an excess incidence of acute myeloid leukaemias and myelodysplastic syndrome to be associated with therapy in those patients who received doxorubicin combined with intensified cyclophosphamide doses (requiring granulocyte colony-stimulating factor support) when compared with those who received doxorubicin combined with cyclophosphamide therapy at the standard dose. In our study, women who received chemotherapy in addition to surgery were excluded.

An excess incidence of lung cancer was observed at 10 years or more after radiotherapy. This is consistent with other studies (Deutsch *et al*, 2003; Zablotska and Neugut, 2003). Smoking is the main cause of lung cancer (Ernster, 1994). The SIRs for lung cancer in both the RT and non-RT cohorts were found to be low, especially at 1–10 years after breast cancer diagnosis. It is possible that the diagnosis of cancer in some of these individuals prompted voluntary smoking cessation with a subsequent decrease in lung cancer incidence, as has previously been observed following smoking cessation (Peto *et al*, 2000). However, information on the smoking status of patients in our study was not available.

It is also of note that, in radiotherapy patients, a significant decrease in the observed incidence of lung cancer was found in the year following diagnosis of breast cancer. This could be due to genuine decreased incidence, underdiagnosis or chance. Underdiagnosis could result from the misinterpretation of the signs and symptoms of lung cancer as side effects of recent breast radiotherapy. If such is the case, then this certainly deems further investigation. If a genuine decrease in incidence of lung cancer results from radiotherapy, it is perhaps possible that small incipient lung tumours responded to breast RT with a subsequent decrease in incidence, though this is unlikely upon consideration of the typical radiotherapy dose distribution.

There was a generally elevated incidence of breast cancer above the standardised baseline in both the RT and non-RT cohorts, consistent with a genetic or environmental predisposition hypothesis. However, the isolated increase in RR in the RT cohort at 5–10 years is most plausibly attributed to a direct effect of RT. In this 5–10-year interval, the proportion of the second breast cancers which were contralateral was the same in the two cohorts (94%). Considering the type of surgery that was performed in this group of patients, 20% of the second breast cancers in the non-RT cohort occurred in women who had initially been treated with a partial removal of the breast, as opposed to radical mastectomy. In the RT cohort, the corresponding figure was significantly higher (42%). This suggests that a larger proportion of women with a radiation-induced second breast cancer had initially had lumpectomy rather than mastecotomy, when compared with the non-RT cohort.

An excess of cancer of the colon following breast cancer radiotherapy has been reported previously (Harvey and Brinton, 1985). However, no significant excess was observed in this study. Similarly, no significant increase in incidence of thyroid cancers was observed in any interval in radiotherapy recipients compared with non-RT recipients, although this may have been due to limited statistical power associated with the general low incidence of this cancer. We found a significant increase in the incidence of oesophageal cancers 15 or more years after radiotherapy. There is increasing use of breast conservation therapy (lumpectomy and radiotherapy) for Stage I and II invasive carcinoma and for ductal carcinoma *in situ*, and evidence exists to support its efficacy when compared with mastectomy alone (Jacobson *et al*, 1995). Deutsch has suggested that this trend, along with renewed enthusiasm for postmastectomy chest wall and regional lymph node irradiation, may lead to increasing numbers of women with breast cancer being exposed to pulmonary irradiation, with possible implications for second cancer incidence (Deutsch *et al*, 2003). However, a study by Zablotska and Neugut (2003) has shown only post-mastectomy radiotherapy, and not post-lumpectomy radiotherapy, to be associated with significantly increased incidence of lung cancers after 10 years. It is uncertain what effect new technologies such as intensity-modulated radiation therapy will have on the incidence of second cancers compared with conventional three-dimensional conformal radiation therapy, although Hall and Wuu (2003) have pointed out that, since the technique requires the use of more fields, a larger volume of surrounding tissues are exposed to a lower dose than in conventional radiotherapy, and since these fields are modulated and therefore require a longer charging time for the energiser, the patient is exposed to a higher level of radiation leakage. By applying a calculated dose–response relationship to dose–volume histograms of radiotherapy by conventional and intensity-modulated methods, the authors have concluded that intensity-modulated radiation therapy may result in a higher incidence of second primary cancers than conventional conformal radiotherapy.

With a fairly low overall incidence of second cancers, the excess risk associated with radiotherapy remains small. In absolute terms, the number of observed excess second cancers in the RT cohort of 33 763 women which can be attributed to radiation exposure is about 160. It is therefore important to note that in breast cancer the benefits of radiotherapy still very much outweigh the risks of developing subsequent second cancers.
